# An attempted “suicide pact” in Covid-19 era – psychiatric perspectives

**DOI:** 10.1186/s12888-022-04333-z

**Published:** 2022-11-04

**Authors:** Benedetta Nardi, Luca Del Prete, Giulia Amatori, Barbara Carpita, Claudia Carmassi, Maurizio Pompili, Liliana Dell’Osso

**Affiliations:** 1 Department of Clinical and Experimental Medicine, University of Pisa, Via Roma 67, 56127 Pisa, Italy; 2 Department of Neurosciences, Faculty of Medicine and Psychology, Mental Health and Sensory Organs, Sapienza University of Rome, Rome, Italy

**Keywords:** Suicide pact, COVID-19, Psychological impact, Suicide, Autism spectrum

## Abstract

**Background:**

A "suicide pact" is a joint and actively induced death of two individuals with the essential and unavoidable characteristic of a mutual consent. One of the partners (dominant in the relationship, commonly male) usually induces the action and in most cases, it is the one who actively carries it out. Undiagnosed psychopathological dimension or pathological subthreshold traits are found in those who enter into suicide agreements, the presence of cluster B personality traits such as narcissistic or borderline is of particular relevance in the dominant partner, while in the submissive one dependent personality traits are more frequent. As in the case of other similar health emergencies, COVID-19 pandemic seems to lead to greater suicidality, including the "suicide pacts" of couples whose motivation varies including firstly financial problems, strictly followed by fear of infection and not being able to return home from abroad.

**Case presentation:**

We reported a case of a couple who entered a suicide agreement consequently to the economic difficulties caused by COVID-19 pandemic, hospitalized in our department. Both partners were assessed with Adult Autism Subthreshold Spectrum (AdAS Spectrum) and both crossed the threshold for clinically relevant autistic traits (M = 67; F = 49).

**Conclusion:**

This case further confirms the link between COVID-19 pandemics and suicidality. The role of autism spectrum traits as a vulnerability factor towards the development of severe psychopathological consequences after traumatic events is also stressed.

## Background

The term "suicide pact" refers to the joint and actively self-induced death of two individuals occurring in the same period of time, in relation to similar reasons and previous mutual consent [1–4]. These acts are often brought to public attention through the news and the media; however, the medical literature is still scarce in this field, especially the one regarding the survivors [4–7]. Previous studies have estimated a frequency of suicide pacts ranging from 0.6% to 4% [1, 3, 4, 8]. Similarities and differences between the various cases of "suicide pacts" have been debated since the 1960s [1, 4, 7], but the essential and unavoidable characteristic is mutual consent [9, 10]. Although this pact is generally carried out by mutual agreement, one of the partners (who is dominant in the relationship, commonly male) usually induces the action and, in most cases, is also the one who actively carries it out [2, 7, 9]. The primary causes of these acts are still widely debated. An undiagnosed psychopathological dimension or pathological subthreshold traits are typically found in those who enter into suicide agreements: in particular, some studies highlighted a prevalence of personality traits typical of cluster B such as narcissistic or borderline personality, in the dominant partner, and of cluster C, such as dependent personality, in the submissive one [2, 9]. The potential risk of a suicide pact may also be predicted based on the anaclitic structure of the sentimental relationship, often characterized by a high degree of mutual social isolation, defined as "encapsulated unity". This type of mutual dependence can to transform any adverse external event (such as the loss of a house, deterioration of the health, or impediments to the relationship by third parties) into a threat to the couple's unity, thus eventually triggering the joint suicide [2, 7, 9]. The more recent “suicide pacts” involving consorts, which have been reported during the COVID-19 pandemic, seem at least in part to feature the above-described pattern [11]. The reasons behind COVID-19-related suicide pacts may include fear of infection, financial problems, and not being able to return home from abroad [11]. In particular, financial problems caused by local and foreign lockdowns have been reported to be the main risk factor, strictly followed by fear of infection [12, 13]. The COVID-19 pandemic caused a severe global financial recession, in addition to the impact on physical health, the current pandemic has also had a huge psychological impact on millions of people around the world [14]. The new disease caused economic, social, and mental crises in a very short time [15]. Moreover, COVID-19’s rapid local and international spread, together with the pervasiveness of the disease, have allowed it to affect several areas of human life, such as physical health, social well-being, financial well-being, etc. All these areas are well known to have a direct link with the balance of mental health [16–20], while numerous shreds of evidences specifically stressed the critical impact that economic and financial crises could exert on psychological vulnerabilities [21–23]. In particular, a systematic review observed how economic distress and suicide rate were clinically correlated in 31 out of 38 studies [22]. Therefore, it may be easily understood how various problems related to the COVID-19 pandemic such as the economic recession and the restriction of movements (e.g. isolation, quarantine, etc.) may have led to different kinds of psychological suffering, such as anger, annoyance, anxiety, fear, frustration, sense guilt, helplessness, loneliness, nervousness, sadness, and worry [18, 20, 24–26]. Furthermore, for individuals with pre-existing psychiatric conditions or vulnerabilities [27, 28], the most extreme consequence of such mental distress may have led to suicidal thoughts, suicide attempts, and/or actual suicide [29, 30]. In the framework of vulnerability towards psychopathology, several studies are stressing the specific importance of autistic traits. Autism spectrum symptoms, also when subthreshold, seem indeed to be linked to a detrimental impact on quality of life, being a significant vulnerability factor for developing other psychiatric conditions and even suicidal thoughts and behaviors, in particular after traumatic or stressful events [31–37]. Moreover, a link between autistic traits and personality disorders has also been stressed [38]. As previous pandemics (e.g.: severe acute respiratory syndrome, SARS, in 2002) [39], the current COVID-19 pandemic has been associated with increased suicidality, including in the forms of "suicide pacts" of couples who died together in a context and motivation related to COVID-19. As a matter of fact, various researches are currently available from all over the world concerning cases of suicide that occurred in the context of the current pandemic [12, 40]. The psychiatric effects of the COVID-19 crisis have become evident in recent years and are likely to continue to materialize in the years to come as the long-term consequences of chronic anxiety, prolonged distress, physical distancing, loneliness, death of friends and family and job loss. In this framework, we reported a case of a couple who entered a suicide agreement consequently to the economic difficulties caused by COVID-19, with a specific focus on the presence of autistic traits as a potential vulnerability factor.

## Case presentation

In the early morning of January 21^st^, 2022, the partners Q.X. and K.Y. accessed the Emergency Department of the Pisan University Hospital after an attempted suicide via intoxication from CO and benzodiazepines. The patients planned the suicide attempt and agreed upon it during the previous week, in association with the worsening of the couple's economic situation. Later in the afternoon, both were transferred: the first at the psychiatric ward of the Pisan University Hospital (AOUP), while the second at the psychiatric ward of the territorial Psychiatric Diagnosis and Care Service (SPDC).

Q.X. is a 60 years old woman, (height: 1.65mt; weight: 72 kg) divorced, living with her current partner K.Y. for the last 20 years. In her medical history, nothing relevant emerged, aside from hypothyroidism treated with levothyroxine 50 mg/day since when she was 26; during the pandemic, she contracted the Covid-19 virus in November 2020 and by the time of our assessment she had been vaccinated with 2 doses of Pfizer/BioNTech Comirnaty vax (in July and August 2021). She graduated from high school, worked for many years in catering and was currently unemployed at the time of the observation due to the recent bankruptcy of the restaurant she ran with her partner. Q.X. has always been described as unselfish, generous and with a strong sense of duty, a perfectionist at work and with a high moral rigor. She also reports from childhood and adolescence to be sensitive to separation, both from parents in childhood and from partners subsequently, sensitive to the judgment of others, not very flexible to unexpected changes in her routine, showing difficulties in making decisions independently and highlighting elements of reward dependence. After graduating from high school, Q.X. interrupted her studies to devote herself to various work activities without ever finding real employment and sentimental stability. At 40, she met her current partner with whom she decided to start her own restaurant.

K.Y. is a 64 years old male (height: 1.85; weight: 79 kg); his medical history reported nothing relevant; during the pandemic, he contracted the Covid-19 virus in November 2020 and by the time of our assessment he had been vaccinated with 2 doses of Pfizer/BioNTech Comirnaty vax (in July and August 2021). K.Y. described himself as a sociable, eccentric, outgoing person, with high levels of energy and a tendency towards novelty seeking, but also highly dedicated to work, with keen attention to detail and a great sense of duty. From the interview at the entrance, histrionic personality traits also emerged, such as a tendency to theatricality, winking and allusive mimicry and a partially devaluing attitude towards the medical and nursing staff. After having graduated from high school, he began his university studies at the faculty of Architecture; however, he was prematurely forced to interrupt his academic career due to the loss of his mother, followed by a serious accident of his father. K.Y. said that he had always coped with psychological stressors and various traumas by focusing and dedicating himself completely to work, first enlisting as a soldier for about a year and then becoming an electrician in a construction company, always managing to maintain an apparently adequate psycho-affective and work adjustment. Shortly after meeting Q.X., he quit his job to open his own business with his partner in catering. None of the patients had a previous history of suicide attempts, nor of any manifested mental disorder; the present is the first time for both of them to be assessed by a Psychiatrist.

The onset of the psychopathological picture is traced back to March 2020, after the worsening of the couple's financial situation. During the previous two years, they had to repeatedly close their business: the first time due to economic and managerial difficulties, after which they moved to a new region, but they had to close their business a second time for logistical problems. They announced the opening of a new business in 2020, thanks to the earnings from the sale of their home and loans received from close family members. However, in March 2020, when they are forced to close temporarily due to the first lockdown after only 8 days from the inauguration, they both developed symptomatology characterized by subclinical mood swings, episodes of emotional lability, with dysphoric cues and secondary demoralization, ruminative thinking focused on economic and work difficulties with an increase of anxious thoughts sharing, internal tension and progressive worsening of the sleep pattern. Over the months, along with the prolongation of the social restrictions and the requested closure of the restaurant, the clinical picture of the patients progressively increased in severity, reaching a peak after the definitive closure of their business on December 31^st^ 2021. In the following month, K.Y. progressively developed feelings of shame and despair, thoughts of ruin and a sense of abandonment towards friends and relatives (“we felt like we were surrounded by darkness”), subsequently shared by his partner. Death ideas and suicidal ideation were also reported, with growing intensity, initially causing a strong conflict with the partner ("I told him it was a madness, we fought a lot about this"). The latter, due to the persistence of K.Y.’s suicidal ideation, developed more severe anxiety symptoms concerning a possible separation and to the thought of a life without the partner (“without him I would not have been able to face the world”). During the week before the hospitalization, the increasing pervasiveness of this condition led to the planning in the last week of repeated suicide attempts via self-cutting, and finally to accurately organize the more lethal attempt that caused the hospitalization. Two days prior to the attempt, K.Y. went to his family doctor reporting that lately he was feeling “a bit anxious and more tense” and asking for “some of the drops that everyone is using”; for this purpose, the referring doctor suggested Delorazepam oral solution 1 mg/ml, 5 drops in case of need (not more than 3 times a day) and 8 to 10 drops before going to sleep. The day prior to the attempt K.Y. proceeded to retrieve a confection said formulation of Delorazepam from a nearby Pharmacy; during our assessment both K.Y. and Q. X. denied past use of Delorazepam or any other kind of Benzodiazepine and psychotropic drugs, they both confirmed the day of the attempted suicide as the first time of using this kind of medicine. In the late evening of January 20^th^, K. Y. proceeded to interrupt the house electricity supply, in order to prevent a possible explosion with the involvement of third parties, and then manumitted the kitchen stove so that they would produce a continuous emission of CO during the night, then administered approximatively 60 drops of Delorazepam (about 2,4 mg) to his wife and to himself before going to bed, also giving the same drug to their dogs, dragged with them in a pseudo-picture of suicide “pietatis causa”. (“It was our act of love” G.M.). No notes were found nor reported by the couple. In the early morning of the following day, both partners woke up with what they called a “throbbing headache”, Q. X. aware of the failure of the attempt and already criticizing the idea, alerted the neighbors who proceeded to call an ambulance and assist them until the arrival of the paramedics. Both dogs were taken to a vet clinic by the neighbors but only one of them managed to survive without complication, the other, a 15 years old Labrador passed away in a vet clinic on January 24^th^ due to respiratory and cardiac complications, no autopsy was performed on the dog.

At the time of the admission Q. X. was vigil, lucid and well oriented, available and accessible for the interview.

The mood was shown to be oriented towards the negative pole, the thinking process was normal in its shape and content. She firmly criticized the suicide attempt carried the night before and verbalized a strong preoccupation concerning the well-being of their dogs and the possibility of being separated from K. Y. for the time of the hospitalization.

At the time of the admission K. Y. was vigil, lucid and well oriented, partially available and accessible for the interview. The mood was shown to be oriented towards the negative pole with some dysphoric cues. The thinking process appeared to be normal in its shape; suicidal ideation and feeling of discouragement and insolvency were still pervasive even if partially sensible to the critics.

**Fig. 1:**
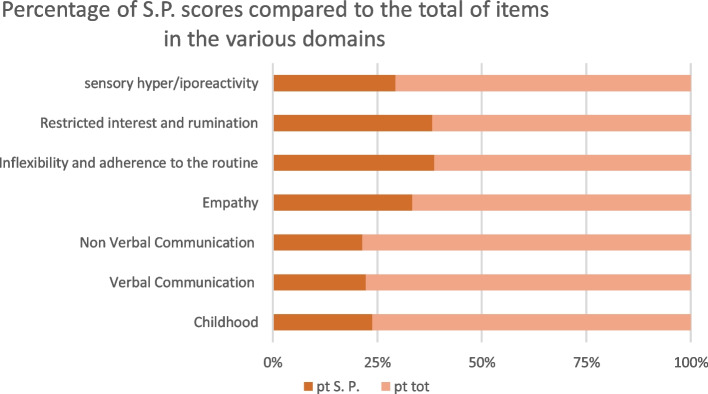
Percentage scores reported by the female partner on AdAS Spectrum domains

**Fig. 2:**
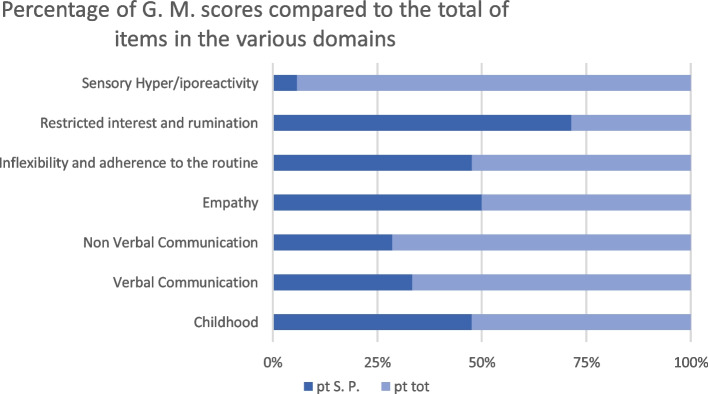
Percentage scores reported by the male partner on AdAS Spectrum domains

During the hospitalization, both patients underwent a clinical examination and were assessed by the Adult Autism Spectrum (AdAS Spectrum) self-assessment questionnaire, tailored for identifying the presence of autistic traits indicative of an underlying psychopathological vulnerability [41]. Q.X. reached a total score of 49 (Fig. 1; Table 1) and K.Y. of 67 (Fig. 2; Table 2). Both scores were above the threshold of the scale indicating the presence of significant autistic traits, while the threshold for the possible presence of a full-blown Autism Spectrum Disorder (ASD) is 70 [42].

**Table 1:** Scores reported by the female partner

Domains	Score	Percentage score
Childhood/adolescence	5	23.80%
Verbal communication	4	22.22%
Non verbal communication	6	21.42%
Empathy	4	33.33%
Inflexibility and adherence to the routine	17	39.53%
Restricted interest and rumination	8	38.09%
Sensory hyper/iporectivity	5	29.41%
Total	49	36.62%

**Table 2:** Scores reported by the male partner

Domains	Score	Percentage Score
Childhood/adolescence	10	47.62%
Verbal communication	6	33.33%
Non verbal communication	8	28.56%
Empathy	6	50%
Inflexibility and adherence to the routine	21	48.84%
Restricted interest and rumination	15	71.43%
Sensory hyper/iporectivity	1	5.88%
Total	67	41.87%

During the hospitalization, both Q.X and K.Y. underwent an electrocardiogram and general blood test, nothing relevant emerged from those exams. Q. X. was treated with a Mood Stabilizer (Carbamazepine tablets, 300 mg twice a day) and an Antidepressant (Sertraline tablets, 50 mg once a day); K. Y. was treated with a Mood Stabilizer (Lithium Carbonate capsule, 300 mg three times a day) and Antipsychotic (Olanzapine tablets, 10 mg once a day.

## Discussion and conclusion

The above-reported case is in line with previous reports of a link between suicidal risk and the COVID-19 pandemic [12, 40]. From a dimensional perspective, subthreshold conditions and traits of vulnerability are of particular importance in determining the risk of worse outcomes. However, these psychopathological conditions often do not reach clinical attention, except in a later moment and with secondary manifestations [31, 43]. Among the other psychopathological spectra, the specific detrimental role of autism related traits in psychopathology has been stressed by several works [31–35, 44]. To date, the current version of the Diagnostic and Statistical Manual of Mental Disorder (DSM-5) still does not take into account the broader spectrum of subthreshold manifestations distributed as a continuum in the non-clinical population [45]. In particular, the relevance of the subthreshold autism spectrum lies in the fact that, according to a large number of studies, not only full-blown ASD, but also milder presentations or even subthreshold autistic traits could actually interfere with general functioning, representing a significant risk factor for the development of suicidal ideation [31, 46, 47]. Taking into account the growing number of studies that highlighted the presence and relevance of autistic traits in clinical populations not affected by full-blown ASD [31–33, 36–38, 48], it has also been suggested the hypothesis that different types of psychiatric disorders may be underlain by an ASD-like neurodevelopmental alteration, whose interaction with the environment and life events may lead to different psychopathological trajectories [44].

Noticeably, the case presented here reported the presence of suicide attempts in two patients with no previously diagnosed psychiatric conditions, and who reported a significant presence of autistic traits. While the COVID-19 pandemic was reported to be a potential risk factor for developing psychopathological conditions, also among subjects who were not directly affected by the infection, but only by the social and economic consequences [18, 20, 24–26], the potentially detrimental effect of stressful events, even if of milder intensity, on the psychopathological trajectory of subjects with autistic traits, have been previously stressed in the literature [34, 35, 44].

This case is also in line with the extensive literature that highlighted a high prevalence of suicide attempts, suicidal thoughts and behaviors among ASD patients or in subjects with autistic traits [31–33, 48–51]. It is worth mentioning that both the patients reported the highest percentage scores on the AdAS Spectrum *Restrictive Interest and Rumination* and in *Inflexibility and adherence to routine* domains. A previous study by Dell’Osso et al. [31] highlighted a significant correlation between all domains of the AdAS Spectrum and suicidality, and in particular how the greatest correlation was reported between suicidality and the AdAS Spectrum domain measuring the presence of narrow interests and rumination. Rumination is a negative pattern of repetitive thinking, usually associated with and exacerbated by anxiety and depression, which often affects problem solving and the processing of negative feelings, leading to progressive social isolation [52–54]. Although ruminative thinking has been described as one of the main symptoms of ASD [55], it could be considered a trans-nosographic dimension, detectable in several kinds of psychiatric disorders and typically associated with a worse outcome [52, 54, 56, 57], possibly playing a role in the development of suicidal ideas and behaviors [34, 49]. Globally, this case seemed to further confirm the link between autistic traits and suicidality risk, as well the increased vulnerability of subjects in the autism spectrum towards stressful life events. In the post-pandemic period, the vulnerability linked to sub-threshold psychopathological conditions may represent a hidden emergency that should be carefully addressed in both research and clinical settings in order to develop adequate strategies for prevention and treatment.

## Limits

This paper reports many limits. In the first place the literature on the association between suicidal thoughts and the COVID-19 pandemic shows various and conflicting results. Certainly, it indicates a great variability based on place, time, and ethnicity of the population in exam, highlighting a increased prevalence of suicidal thoughts in the non-white population and not in the white. Results regarding the Italian population are still scarce and contradictory.

A second limit is the lack of information regarding some of the aspects of the event: for instance, we were not able to retrieve information on the lot number and expiration date of the confection of Delorazepam used, toxicology exams were not performed on the couple, and no autopsy were performed on the deceased dog, nor toxicology exams were performed on the one who survived.

Lastly, we recognize that many other factors, aside from ASD, were not assessed and could have played a role as precipitating factors in the developing of the described situation and furthermore that the cessation of the job may not be the only associated factor in the scope of external events even independently of how it was reported to us from the patients.

## Data Availability

All data generated or analyzed during this study are included in this published article.

## Abbreviations

AdAS Spectrum: Aduls Autism Subthreshold Spectrum
COVID-19: Coronavirus Disease 2019
SARS: Severe acute respiratory syndrome
AOUP: Pisan University Hospital
SPDC: Psychiatric Diagnosis and Care Service
ASD: Autism Specrtum Disorder
DSM-5: Diagnostic and Statistical Manual of Mental Disorders, Fifth Edition
